# Anatomical Heterogeneity in Longitudinal CA15-3 and CEA Rises Preceding Metastatic Breast Cancer Progression

**DOI:** 10.3390/ijms27198502

**Published:** 2026-09-23

**Authors:** Zsanett Szilvási, Borbála Bruszniczky, János Tibor Fekete

**Affiliations:** 1 Department of Bioinformatics, Semmelweis University, Tűzoltó utca 7–9, H-1094 Budapest, Hungary; bende.zsanett@phd.semmelweis.hu (Z.S.); brusznibori@gmail.com (B.B.); 2 Institute of Transdisciplinary Discoveries, University of Pécs Medical School, Szigeti Street 12, H-7624 Pécs, Hungary; 3 Institute of Molecular Life Sciences, HUN-REN Research Centre for Natural Sciences, Magyar tudósok körútja 2, H-1117 Budapest, Hungary

**Keywords:** metastatic breast cancer, CA15-3, CEA, tumor markers, disease progression, longitudinal monitoring, real-world data, biomarker surveillance

## Abstract

Cancer antigen 15-3 (CA15-3) and carcinoembryonic antigen (CEA) are used alongside clinical and radiologic assessment in metastatic breast cancer, but progression may occur without a preceding increase in either marker. We examined whether this differed according to newly documented anatomical involvement around radiographic progression. We analyzed 4517 radiographic progression events from 1592 patients in the Memorial Sloan Kettering–Clinical History of Real-World Data (MSK-CHORD) breast cancer cohort. A qualifying marker rise was defined as a ≥25% increase in CA15-3 or CEA from the preceding running nadir, occurring more than 30 days after treatment-line initiation and within 90 days before progression. Anatomical associations were estimated using patient-clustered logistic generalized estimating equations. A total of 1650 events (36.5%) occurred without a preceding qualifying rise. This proportion ranged from 24.8% for newly documented liver involvement to 45.1% for central nervous system involvement. Liver involvement was associated with lower odds of progression without a preceding rise (adjusted OR 0.53, 95% CI 0.40–0.71), whereas central nervous system involvement was associated with higher odds (OR 1.60, 95% CI 1.19–2.14). The associations remained consistent across sensitivity analyses. Thus, among documented radiographic progression events, the frequency of a preceding CA15-3 or CEA rise differed according to newly documented anatomical involvement.

## 1. Introduction

Serum cancer antigen 15-3 (CA15-3) and carcinoembryonic antigen (CEA) are commonly measured during the follow-up of metastatic breast cancer. Current American Society of Clinical Oncology (ASCO) guidance considers CA15-3 and CEA as adjunctive assessments in metastatic breast cancer and recommends that treatment decisions should not be based on these markers alone [1]. Contemporary European Society for Medical Oncology (ESMO) guidance provides the broader framework for diagnosis, treatment, and follow-up of metastatic breast cancer [2]. Nevertheless, serial CA15-3 and CEA measurements are frequently available in routine practice and are often interpreted together with imaging. A rising marker may support suspicion of disease progression, but the clinical meaning of stable values is less certain.

CA15-3 and CEA concentrations vary considerably, according to the extent and characteristics of metastatic disease. Previous studies have reported associations with metastatic burden, molecular subtype, and metastatic distribution, with differences particularly described for liver and bone involvement [3,4,5,6,7]. Wang et al. found differences in serum-marker positivity according to metastatic site [3], while more recent studies have confirmed associations between CA15-3 levels and the presence or extent of metastatic disease [5,6]. However, not all studies have demonstrated consistent site-specific differences [7]. Most of this evidence concerns marker concentrations at metastatic diagnosis or recurrence rather than changes during subsequent treatment.

Serial changes in CA15-3 and CEA have also been studied during follow-up and systemic therapy [8,9,10,11]. Sottotetti et al. reported increases in both markers between best radiologic response and subsequent progression in patients with metastatic breast cancer treated with endocrine therapy and cyclin-dependent kinase 4/6 (CDK4/6) inhibition [9], while Laessig et al. examined CA15-3 and CEA across successive progression events in metastatic disease [10]. Anatomical differences have also been reported in the recurrence setting. De Cock et al. found that CA15-3 elevation was the first sign of secondary metastatic breast cancer in 45% of patients with liver metastases but only 7% of those with brain metastases [11]. These findings suggest that the information provided by serum markers may depend partly on metastatic site. They do not, however, establish whether an individual radiographic progression event during established metastatic treatment is more or less likely to be preceded by a CA15-3 or CEA rise, according to the organ involved. A marker that is elevated in a patient with metastatic disease may not necessarily increase further when that disease progresses.

Evaluation of this question in routine clinical data also depends on the timing of marker measurements and imaging. When marker measurements are sparse or distant from the progression date, absence of a recorded rise may simply reflect that a change was not observed. The timing of imaging similarly determines when radiographic progression becomes detectable. Such observation patterns can bias analyses of longitudinal clinical data [12,13].

We, therefore, analyzed treatment-line-aligned CA15-3 and CEA measurements and radiographic progression events in the Memorial Sloan Kettering–Clinical History of Real-World Data (MSK-CHORD) breast cancer cohort. We examined whether newly documented liver, bone, lung/pleural, central nervous system (CNS), or other visceral involvement was associated with progression occurring without a preceding marker rise, restricting the primary analysis to events with adequate longitudinal marker surveillance.

## 2. Results

### 2.1. Study Cohort and Progression Events

The treatment-line dataset contained 19,822 records. Of these, 8083 metastatic treatment lines had radiographically documented progression, and 6685 remained after excluding progression within 30 days of treatment initiation. Tumor-site information within ±30 days of progression was available for 6268 events, and adequate longitudinal surveillance of both CA15-3 and CEA was available for 4774 events. A total of 4517 events met both criteria and were included in the primary analysis. These 4517 events were contributed by 1592 patients (Figure 1). At least one newly documented organ site was identified for 887 events (19.6%); the remaining 3630 events (80.4%) had none of the five newly documented organ indicators. Among the 6268 site-covered eligible progression events, 1751 were excluded because adequate dual-marker surveillance was unavailable. Differences between included and surveillance-excluded events were modest across measured characteristics, with absolute standardized mean differences ≤ 0.196 (Supplementary Table S9A,B). The 1592 patients in the primary cohort contributed a median of two progression events (interquartile range [IQR] 1–4; range 1–15); 33.9% contributed one event, 22.2% two, 14.6% three, and 29.3% four or more events (Supplementary Table S9C). A qualifying CA15-3 or CEA rise preceded 2867 events (63.5%), whereas 1650 (36.5%) occurred without a preceding rise.

Most progression events occurred in patients with hormone receptor-positive/human epidermal growth factor receptor 2-negative (HR+/HER2−) disease (n = 3301), followed by HER2-positive (HER2+) (n = 753) and triple-negative breast cancer (TNBC) (n = 463). Chemotherapy was the most frequent treatment class (n = 2122). Baseline values for both CA15-3 and CEA were available for 3915 events (Table 1).

### 2.2. Anatomical Heterogeneity in Preceding Tumor-Marker Rises

The proportion of progression events without a preceding CA15-3/CEA rise differed according to newly documented metastatic involvement (Figure 2; Table 2). It was lowest for liver involvement at 24.8% (71/286), followed by lung/pleura at 30.9% (60/194), other visceral sites at 34.4% (62/180), bone at 43.7% (66/151), and CNS at 45.1% (93/206).

In the primary generalized estimating equations (GEE) model, the five anatomical indicators were jointly associated with the absence of a preceding marker rise (global Wald χ^2^ = 35.07, 5 degrees of freedom; *p* = 1.46 × 10^−6^). Newly documented liver involvement was associated with lower odds (adjusted odds ratio [OR] 0.53, 95% confidence interval [CI] 0.40–0.71; Benjamini–Hochberg false-discovery rate [BH-FDR] *p* < 0.001), whereas CNS involvement was associated with higher odds (OR 1.60, 95% CI 1.19–2.14; BH-FDR *p* = 0.004). Bone involvement was not statistically associated with progression without a preceding marker rise (OR 1.39, 95% CI 0.98–1.96), while lung/pleural and other visceral involvement were not independently associated after multiplicity adjustment (Figure 3A; Table 3).

Marker-sampling intensity and measurement timing were also associated with progression without a preceding rise. Every two additional CA15-3/CEA measurements during the 90 days before progression were associated with lower odds of progression without a preceding rise (OR 0.81, 95% CI 0.75–0.87), whereas each additional week in the mean interval from the most recent CA15-3 and CEA measurements to progression was associated with higher odds (OR 1.11, 95% CI 1.07–1.15; both *p* < 0.001).

### 2.3. Single-Organ and Robustness Analyses

The single-new-organ analysis included 775 events from 639 patients. Compared with events in which the liver was the only newly documented organ site, events in which bone was the only newly documented organ site had higher odds of progression without a preceding marker rise (adjusted OR 2.56, 95% CI 1.51–4.35; *p* = 0.00052), as did events in which CNS was the only newly documented organ site (adjusted OR 2.83, 95% CI 1.78–4.51; *p* = 1.24 × 10^−5^). Lung/pleural and other visceral involvement did not differ significantly from the liver (Figure 3B; Supplementary Table S1).

The anatomical association was robust to alternative definitions. Global organ-site tests remained significant across all combinations of 20%, 25%, and 50% marker-rise thresholds and 60-, 90-, 120-, and 180-day warning windows (all *p* ≤ 6.2 × 10^−5^). The results were also preserved using ±14-, ±30-, and ±45-day windows for assignment of newly documented organ involvement (all *p* < 1.3 × 10^−5^) (Figure 4; Supplementary Table S2). The anatomical association also remained robust when newly documented organ involvement was restricted to first documentation occurring from 30 days before through the progression date, thereby excluding post-progression site documentation (global *p* = 1.99 × 10^−6^). Under this directional definition, the adjusted OR was 0.55 (95% CI 0.41–0.74) for liver involvement and 1.92 (95% CI 1.30–2.82) for CNS involvement (Supplementary Table S2). Within the original ±30-day window, first documentation after the progression date occurred in 11.5% of liver, 11.9% of bone, 17.5% of lung/pleural, 43.2% of CNS, and 15.6% of other visceral assignments; the complete before/day-0/after distributions are provided in Supplementary Table S7.

### 2.4. Influence of Biochemical and Radiologic Surveillance

Restricting the analysis to 3673 events with both markers measured within 30 days before progression produced similar estimates for liver (OR 0.49, 95% CI 0.36–0.69) and CNS involvement (OR 1.58, 95% CI 1.15–2.17).

Adjustment for radiologic assessment density in the full primary cohort also had little effect: the liver OR changed from 0.534 to 0.533, and the CNS OR from 1.598 to 1.637. The global anatomical association remained highly significant (*p* = 1.18 × 10^−6^).

The interval from the last non-progressive radiologic assessment to progression was available for 2137 events. In this subset, the global anatomical association remained significant after adjustment (*p* = 0.025). The liver estimate remained similar (OR 0.57, 95% CI 0.36–0.89), whereas the CNS estimate was attenuated to OR 1.47 (95% CI 0.90–2.41).

In a further sensitivity analysis restricted to 3915 events with both baseline CA15-3 and CEA available, and additionally adjusted for baseline metastatic burden, liver involvement remained associated with lower odds (OR 0.55, 95% CI 0.40–0.76), whereas bone (OR 1.68, 95% CI 1.16–2.45) and CNS involvement (OR 1.68, 95% CI 1.23–2.30) were associated with higher odds (Figure 4; Supplementary Table S3). The baseline CA15-3 was available for 3944 events (median 60 U/mL, IQR 24–221.25), and the baseline CEA was available for 4001 events (median 7.7 ng/mL, IQR 3.1–33.0) (Supplementary Table S8A). Among the 2867 events with a qualifying ≥ 25% rise, confirmation was assessable in 1607, of which 1441 (89.7%) remained at least 25% above the original reference nadir at the next same-marker measurement (Supplementary Table S8B). In the resulting 3257-event confirmation-based sensitivity analysis, the anatomical association remained significant (global Wald *p* = 7.21 × 10^−6^), with ORs of 0.56 (95% CI 0.40–0.78) for liver, 1.96 (95% CI 1.26–3.06) for bone, and 1.83 (95% CI 1.26–2.66) for CNS involvement (Supplementary Table S3). Inverse-probability weighting for surveillance-related selection produced similar results (global Wald *p* = 2.59 × 10^−6^), with an OR of 0.53 (95% CI 0.40–0.70) for liver involvement and 1.53 (95% CI 1.14–2.05) for CNS involvement (Supplementary Table S3). The propensity-score overlap was good, and the truncated stabilized weights showed limited dispersion (range 0.84–1.33; effective sample size 4473; Supplementary Table S9D).

### 2.5. Marker-Specific and Subgroup Analyses

Anatomical heterogeneity was observed for both markers individually (global organ-site *p* = 1.35 × 10^−4^ for CA15-3 and *p* = 5.82 × 10^−5^ for CEA). Liver involvement was associated with lower odds of lacking a rise in both CA15-3 (OR 0.61, 95% CI 0.47–0.79) and CEA (OR 0.64, 95% CI 0.50–0.82). In CEA-specific models, lung/pleural involvement was also associated with lower odds (OR 0.71, 95% CI 0.53–0.96; BH-FDR *p* = 0.040), whereas CNS involvement was associated with higher odds (OR 1.47, 95% CI 1.10–1.98; BH-FDR *p* = 0.025). Smoking adjustment did not materially change the CEA estimates (Figure 5; Supplementary Table S4).

There was no evidence of effect modification by the molecular subtype (global organ-by-subtype interaction *p* = 0.718) or major treatment class (organ-by-treatment interaction *p* = 0.292) (Supplementary Table S5).

### 2.6. Interval from Qualifying Marker Rise to Radiographic Progression

Among the events with a qualifying biochemical signal within the prespecified 90-day window, the median interval from the first qualifying marker rise to radiographic progression was 35 days for liver, 32 days for bone, and 35 days for lung/pleura, CNS, and other visceral involvement. Site-specific bootstrap confidence intervals substantially overlapped (Supplementary Table S6). Because the qualifying rises were restricted to this 90-day window by design, these values should not be interpreted as estimates of clinical lead time.

## 3. Discussion

In this analysis of metastatic breast cancer, the likelihood of a CA15-3 or CEA rise before radiographic progression differed substantially according to newly documented anatomical involvement. More than one-third of adequately monitored progression events occurred without a qualifying increase in either biomarker. This proportion was lowest with liver involvement and highest with CNS involvement. In adjusted models, liver involvement was associated with lower odds of progression without a preceding marker rise, whereas CNS involvement was associated with higher odds. The contrast was larger in the single-organ analysis and remained consistent across alternative marker thresholds, time windows, and surveillance definitions.

Previous studies have linked CA15-3 and CEA concentrations to metastatic burden, molecular subtype, and metastatic site, although findings for individual organs have varied [3,4,5,6,7]. Bone involvement, for example, has often been associated with elevated CA15-3 [3,14]. In our study, however, progression events with newly documented bone involvement were relatively often not preceded by a further qualifying rise, and bone as the only newly documented organ site was associated with higher odds of lacking a preceding rise than the liver as the only newly documented organ site. Marker elevation in established metastatic disease is, therefore, not equivalent to a new marker increase when that disease subsequently progresses.

The liver pattern was more concordant with earlier work. Wang et al. reported higher CA15-3 positivity with liver metastases [3], and De Cock et al. found that increasing CA15-3 was the first sign of secondary metastatic disease in 45% of patients with liver metastases but only 7% of those with brain metastases [11]. We observed a similar anatomical contrast during the progression of established metastatic disease, using treatment-line-aligned measurements of both CA15-3 and CEA. The liver association remained after adjustment for baseline marker concentrations and metastatic burden, making a simple baseline-level explanation less likely.

Progression events with newly documented CNS involvement showed the opposite pattern, with approximately 45% of adequately monitored events occurring without a preceding qualifying rise. The blood–brain and blood–tumor barriers may reduce the transfer of tumor-derived analytes into the systemic circulation [15,16], providing one possible explanation for the lower frequency of preceding serum-marker rises observed among events with newly documented CNS involvement. These mechanisms could not be examined directly in the present dataset. CNS progression may also occur while extracranial disease remains controlled, further limiting the systemic marker signal.

Measurement timing was also important. More frequent marker sampling increased the opportunity to observe a qualifying rise, whereas a longer mean interval from the most recent CA15-3 and CEA measurements to progression was associated with higher odds of progression without one. Similar problems arise when imaging is performed at clinically determined intervals rather than on a fixed schedule [12,13]. Adjustment for radiologic assessment density left the liver and CNS estimates essentially unchanged. In the subset with a documented last non-progressive scan, however, adjustment for the imaging interval attenuated the CNS estimate, suggesting that earlier radiologic recognition may contribute to the observed CNS pattern. Importantly, the CNS association was not explained by post-progression organ assignment: when newly documented involvement was restricted to first documentation on or before the progression date, the CNS association remained significant and increased in magnitude. Nevertheless, CNS involvement differed from the other organ groups in the timing of first documentation, with a substantially larger proportion first recorded after the progression date.

CA15-3 and CEA showed related but not identical anatomical associations. Liver involvement was associated with lower odds of progression without a preceding rise for both markers, whereas the CNS and lung/pleural associations were more evident for CEA. This is consistent with previous work showing that CA15-3 and CEA provide overlapping but complementary information in metastatic breast cancer [4,9,10]. Smoking adjustment did not materially change the CEA-specific estimates. Among events with a qualifying rise within the prespecified 90-day window, the median marker-rise-to-progression interval was approximately one month across anatomical sites. Because this interval was bounded by the study definition, it should not be interpreted as the clinical lead time of CA15-3 or CEA or compared directly with lead times reported in recurrence surveillance [8,17].

CA15-3 and CEA should remain adjuncts to clinical and radiologic assessment rather than substitutes for imaging [1,2]. The present study did not compare marker-triggered with scheduled imaging and cannot determine whether earlier imaging based on marker changes improves clinical outcomes; any implications for surveillance strategies should, therefore, be considered hypothesis-generating. The present progression-event-based analysis does not quantify the probability of progression when tumor-marker values remain stable. The observed anatomical differences, therefore, describe the heterogeneity in the frequency of preceding qualifying marker rises among documented progression events and should not be interpreted as differences in the clinical reassurance provided by stable marker values. We found no evidence that the anatomical pattern differed substantially by molecular subtype or major treatment class, although these observational analyses were not designed to estimate treatment-specific causal effects on marker release.

The large number of radiographically documented progression events and the treatment-line-based longitudinal design are strengths of this study. Marker changes were evaluated relative to preceding same-marker running nadirs, progression without a marker rise was classified only when adequate dual-marker surveillance was available, and repeated events within patients were handled using patient-clustered models. This study also has limitations. Laboratory testing and imaging followed routine clinical practice, so residual differences in observation cannot be excluded. In particular, the overall number of radiologic assessments does not capture imaging modality, anatomical coverage, or symptom-driven, organ-specific ascertainment, which may be especially relevant for CNS involvement. Requiring adequate dual-marker surveillance may also have introduced selection into the analytical cohort, although inverse-probability weighting yielded similar liver and CNS estimates. The tumor-site timeline identifies newly documented organ involvement around progression but does not establish lesion-level progression of a specific pre-existing metastasis. The interval from the last non-progressive scan was unavailable for approximately half of the primary cohort, and non-malignant determinants of CA15-3 and CEA were not comprehensively captured. Assay-specific reference ranges and platform information were also unavailable, precluding a sensitivity analysis based on the upper limit of normal (ULN). Finally, the analysis used a single real-world data resource and requires external validation before the site-specific estimates are used to guide surveillance strategies.

The occurrence of longitudinal CA15-3 and CEA rises before radiographic progression differed according to newly documented anatomical involvement. Among documented progression events, qualifying marker rises were more frequent when liver involvement was newly documented and less frequent when CNS involvement was newly documented. These findings characterize tumor-marker behavior among documented progression events and do not estimate the probability of progression in the absence of a marker rise.

## 4. Materials and Methods

### 4.1. Study Design and Data Source

We analyzed the MSK-CHORD breast cancer cohort obtained from cBioPortal [18,19,20]. The patient-level clinical data were linked to treatment-line information, serial CA15-3 and CEA measurements, radiologic progression assessments, and longitudinal tumor-site records. The unit of analysis was the eligible radiographic progression event, with at most one progression event contributed by each metastatic treatment line; patients could contribute events from multiple treatment lines. The overall study workflow is shown in Figure 1.

### 4.2. Treatment Lines and Radiographic Progression

Treatment-line assignments were taken directly from the MSK-CHORD treatment dataset. Metastatic onset was defined as the earlier of the first documented stage IV diagnosis and the first radiologically documented distant metastatic site. Treatment lines starting on or after this date were classified as metastatic. Lines starting within 30 days before the first documented metastatic date were classified as probable metastatic lines and were also included.

For each treatment line, we identified the first radiographically documented progression occurring after treatment initiation and no later than the start of the subsequent treatment line. Progression recorded on the treatment-line start date or within the first 30 days of treatment was excluded. Each treatment line, therefore, contributed at most one progression event.

### 4.3. Newly Documented Metastatic Involvement Around Progression

Five metastatic site groups were considered: liver, bone, lung/pleura, CNS/brain, and other visceral sites; the latter comprised adrenal gland and intra-abdominal involvement. A site was classified as newly involved when its first radiologic documentation occurred within 30 days before or after radiographic progression. More than one site could be assigned to the same event.

The tumor-site timeline records the first documentation of organ involvement rather than lesion-level progression; these variables, therefore, indicate newly documented involvement around the time of progression, rather than progression of a specific pre-existing lesion. The sensitivity analyses used 14- and 45-day windows. An additional directional sensitivity analysis restricted newly documented organ involvement to the first radiologic documentation occurring from 30 days before through the progression date (−30 to 0 days), thereby excluding sites first documented after progression. Within the primary ±30-day window, first documentation was also categorized as occurring before progression (−30 to −1 days), on the progression date (day 0), or after progression (+1 to +30 days). A separate analysis was restricted to events with exactly one newly documented organ site. In this analysis, the organ site was modeled as a mutually exclusive categorical variable with liver as the reference category, using the same covariate adjustment as in the primary model.

Events with adequate tumor-site coverage but without newly documented involvement in any of the five organ groups remained in the primary analysis, with all five anatomical indicators coded as absent. These events may nevertheless have represented progression of previously documented metastatic disease.

### 4.4. CA15-3 and CEA Assessment

CA15-3 and CEA measurements from 30 days before treatment-line start through radiographic progression were evaluated separately. Same-day duplicate measurements were collapsed to a single patient-day-marker record; days with conflicting same-day values were excluded from the longitudinal calculations. For each valid same-marker measurement, the preceding running nadir was defined as the lowest earlier valid measurement in this interval. A qualifying biochemical signal was defined as a ≥25% increase from this preceding running nadir, occurring more than 30 days after treatment-line initiation and within the 90 days before radiographic progression. Measurements obtained on the day of radiographic progression were not considered preceding signals. A progression event was classified as having a preceding qualifying marker rise when either CA15-3 or CEA met this criterion. Marker units were provided in the source test labels (CA15-3, U/mL; CEA, ng/mL), whereas assay-specific reference ranges and platform identifiers were unavailable. In a confirmation-based sensitivity analysis, a qualifying ≥25% rise was considered confirmed when the next valid same-marker measurement before progression remained at least 25% above the original reference nadir; qualifying-rise events without a subsequent measurement were excluded from this analysis.

Progression without a preceding marker rise was defined only when adequate longitudinal surveillance was available for both markers. For each marker, adequate longitudinal surveillance required at least two valid measurements from 30 days before treatment-line start through progression, including at least one measurement after treatment initiation and before progression and at least one measurement within 60 days before progression. Events without adequate dual-marker surveillance were excluded from classification of the primary outcome. A stricter sensitivity analysis applied the same criteria but required at least one measurement of each marker within 30 days before progression.

Tumor-marker surveillance intensity was defined as the total number of valid CA15-3 and CEA measurements obtained during the 90 days before radiographic progression. The timing of the most recent marker measurements was defined as the mean number of days from the most recent pre-progression CA15-3 and CEA measurements to radiographic progression. In the regression models, the total marker-measurement count was scaled per two additional measurements, and the mean time from the most recent CA15-3 and CEA measurements to progression was scaled per one-week increase. Radiologic surveillance was assessed using the number of distinct radiologic assessment days during the preceding 90 days, excluding the index progression assessment, and, when available, the interval between the last non-progressive radiologic assessment and progression.

### 4.5. Clinical Variables

The molecular subtype was classified as HR+/HER2−, HER2+, or triple-negative breast cancer. The treatment class was derived from systemic antitumor therapies initiated during the first 30 days of each treatment line using a hierarchical classification. For the primary model, the treatments were categorized as chemotherapy, endocrine therapy without CDK4/6 inhibition, endocrine therapy plus a CDK4/6 inhibitor, HER2-targeted non-ADC therapy, ADC, or other systemic therapy. The treatment-line group was categorized as 1, 2, 3, 4, or ≥5. In the complete-case sensitivity analyses, the baseline CA15-3 and CEA were defined as the closest valid measurements obtained from 30 days before through the treatment-line start date and were modeled after log(1 + x) transformation. The baseline metastatic burden was defined as the number of mapped distant-organ groups documented radiologically during the 90 days before or on treatment-line start. Smoking status, categorized as never, former/current smoker, or unknown, was additionally included in the CEA-specific sensitivity models.

### 4.6. Statistical Analysis

The primary outcome was whether radiographic progression occurred without a preceding qualifying rise in either CA15-3 or CEA. Associations with anatomical involvement were estimated using logistic generalized estimating equations with patient-level clustering, an exchangeable working-correlation structure, and robust standard errors.

Because the analytical cohort was conditioned on radiographically documented progression, the analysis estimated the occurrence or absence of a preceding qualifying marker rise among the documented progression events. It was not designed to estimate the probability of progression, conditional on stable marker values, sensitivity, negative predictive value, or the degree of clinical reassurance provided by the absence of a marker rise.

The primary model included the five non-mutually exclusive organ-site indicators and adjusted for the molecular subtype, treatment class, treatment-line group, the total number of valid CA15-3 and CEA measurements during the preceding 90 days, and the mean time from the most recent pre-progression CA15-3 and CEA measurements to radiographic progression. The overall anatomical association was assessed using a five-degree-of-freedom Wald test. Site-specific adjusted odds ratios (ORs) with 95% confidence intervals (CIs) were reported. Accordingly, ORs greater than 1 indicated higher odds that a documented progression event occurred without a preceding qualifying marker rise, rather than a higher risk of disease progression itself. Benjamini–Hochberg false-discovery-rate correction was applied across the five organ-site coefficients in the primary model and separately across the five organ-site coefficients within each marker-specific model.

The sensitivity analyses examined alternative marker-rise thresholds (20% and 50%), warning windows (60, 120, and 180 days), alternative organ-assignment windows, including a directional −30-to-0-day definition excluding post-progression site documentation, stricter marker-surveillance criteria, radiologic surveillance, baseline marker levels and metastatic burden, a confirmation-based ≥25% rise definition within the primary 90-day window, inverse-probability weighting for surveillance-related selection, and analyses restricted to a single newly documented organ site. For the inverse-probability-weighted analysis, stabilized weights for inclusion in the primary analytical cohort were estimated among the 6268 site-covered eligible progression events using the molecular subtype, treatment class, treatment-line group, baseline metastatic burden, the five organ indicators, and pre-index radiology assessment density, and were truncated at the 1st and 99th percentiles. Additional sensitivity analyses used an independence working-correlation structure and incorporated the dataset-level current-age variable. CA15-3 and CEA were also analyzed separately. Organ-by-subtype interactions were evaluated using Wald tests. Organ-by-treatment interaction analyses were restricted to chemotherapy, endocrine therapy without CDK4/6 inhibition, endocrine therapy plus CDK4/6 inhibition, and HER2-targeted non-ADC therapy. The antibody–drug conjugate (ADC) was excluded from the formal interaction model because the liver-by-ADC cell contained no events without a preceding marker rise, resulting in complete separation.

Among progression events with a qualifying biochemical signal within the prespecified 90-day window, the interval from the first qualifying marker rise to radiographic progression was calculated and summarized by organ site. Confidence intervals for median intervals were estimated using 1000 patient-cluster bootstrap samples. All of the analyses were performed in R version 4.3.2 [21]. The generalized estimating equation models were fitted using the geepack package version 1.3.13 [22]. All of the tests were two-sided.

## Figures and Tables

**Figure 1:**
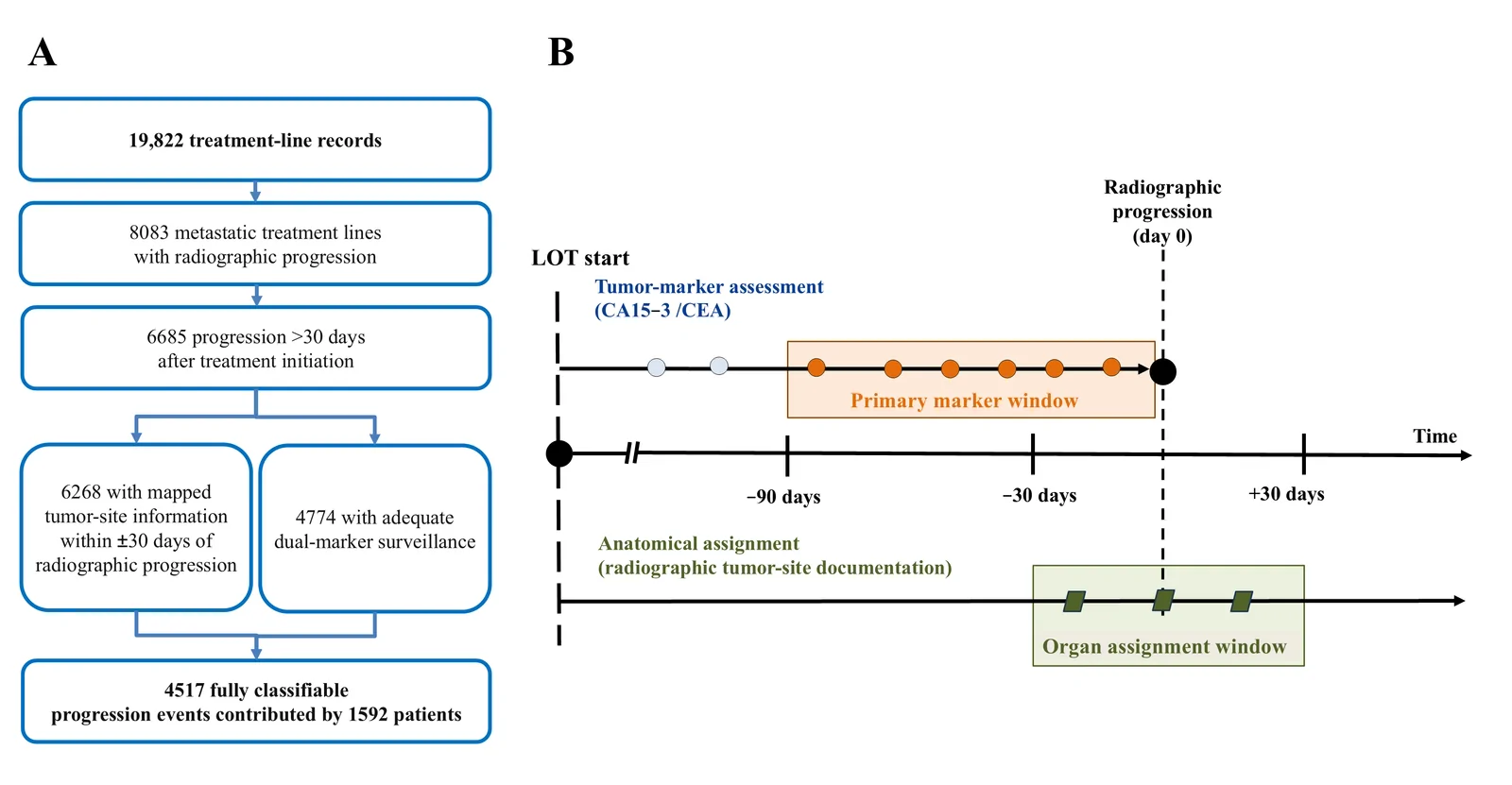
The study cohort derivation and temporal framework for tumor-marker and anatomical assessment. (**A**) The derivation of the primary analytical cohort. Of 19,822 treatment-line records, 6685 metastatic treatment lines had radiographic progression > 30 days after treatment initiation. Among these, 6268 had mapped tumor-site information within ±30 days of progression, and 4774 met adequate dual-marker surveillance criteria; 4517 events from 1592 patients met both criteria. (**B**) The temporal framework. Same-marker running nadirs were constructed using measurements from 30 days before treatment-line start through progression. Qualifying rises occurred >30 days after treatment initiation and within 90 days before progression; day-0 measurements were excluded. Newly documented organ involvement was assigned within ±30 days of progression. Abbreviations: LOT, line of therapy; CA15-3, cancer antigen 15-3; and CEA, carcinoembryonic antigen.

**Figure 2:**
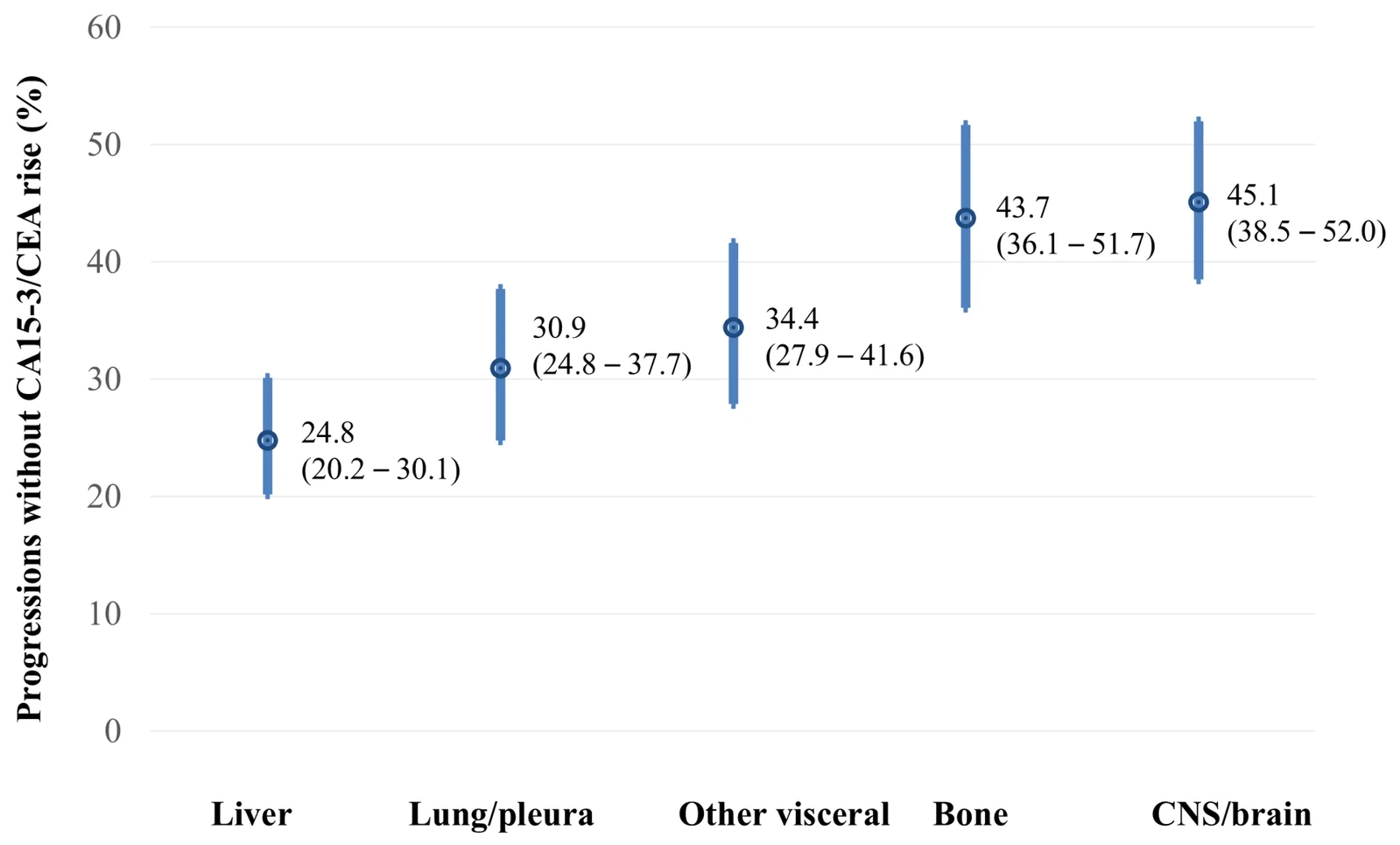
Proportion of radiographic progression events without a preceding qualifying CA15-3 or CEA rise according to newly documented metastatic organ involvement. Points show proportions and vertical bars indicate Wilson 95% confidence intervals among events with adequate dual-marker surveillance. Newly documented organ involvement was assigned within ±30 days of progression; organ-site categories were not mutually exclusive.

**Figure 3:**
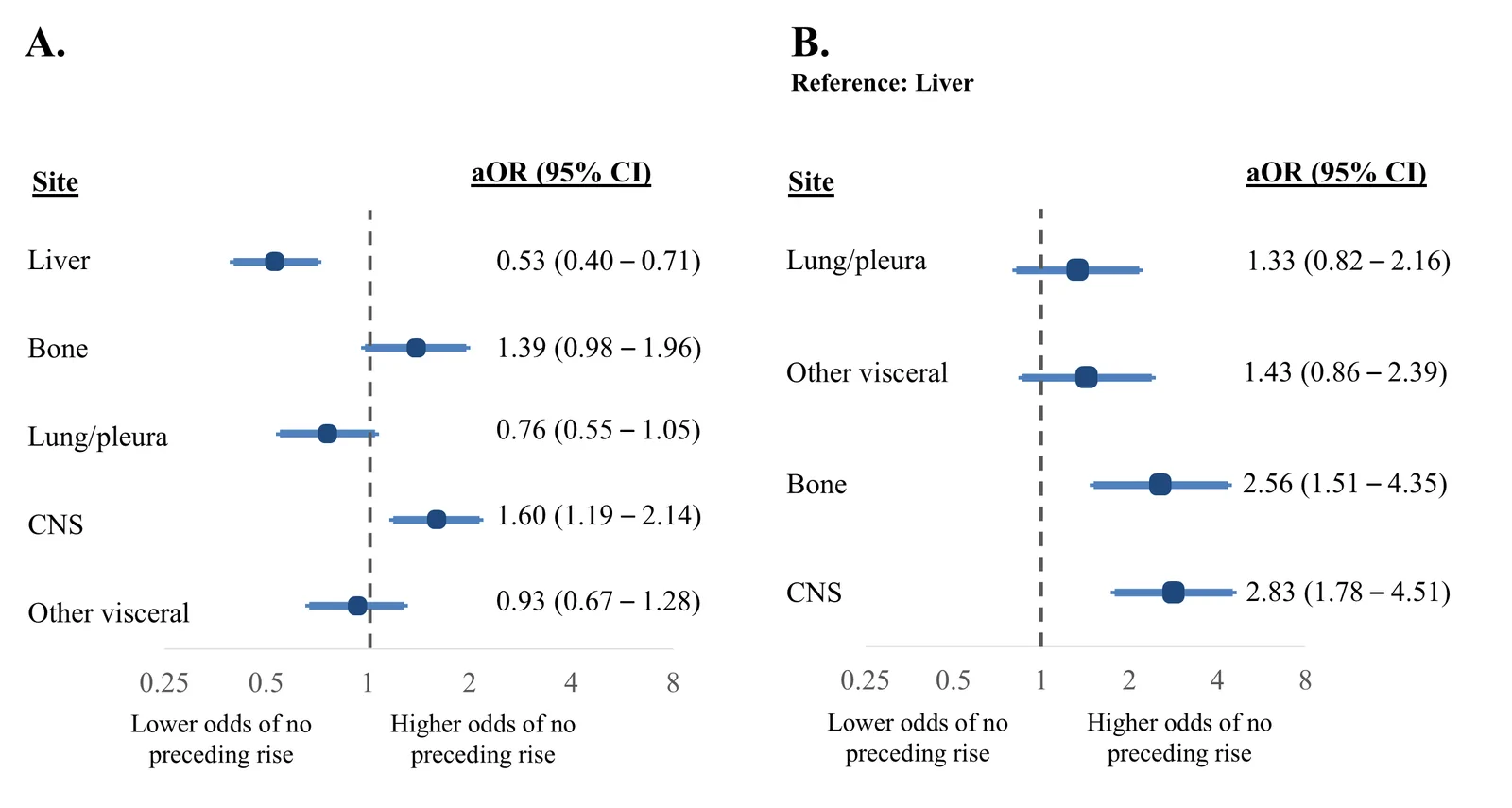
The adjusted associations between newly documented metastatic organ involvement and radiographic progression without a preceding qualifying CA15-3 or CEA rise. (**A**) The adjusted odds ratios (aORs) from the primary patient-clustered logistic GEE model, including all five organ-site indicators simultaneously. (**B**) The sensitivity analysis restricted to events with exactly one newly documented organ site, with the liver as the reference category. The models used the same adjustment set as the primary analysis. The points indicate aORs and horizontal lines indicate 95% confidence intervals; the dashed line indicates aOR = 1.

**Figure 4:**
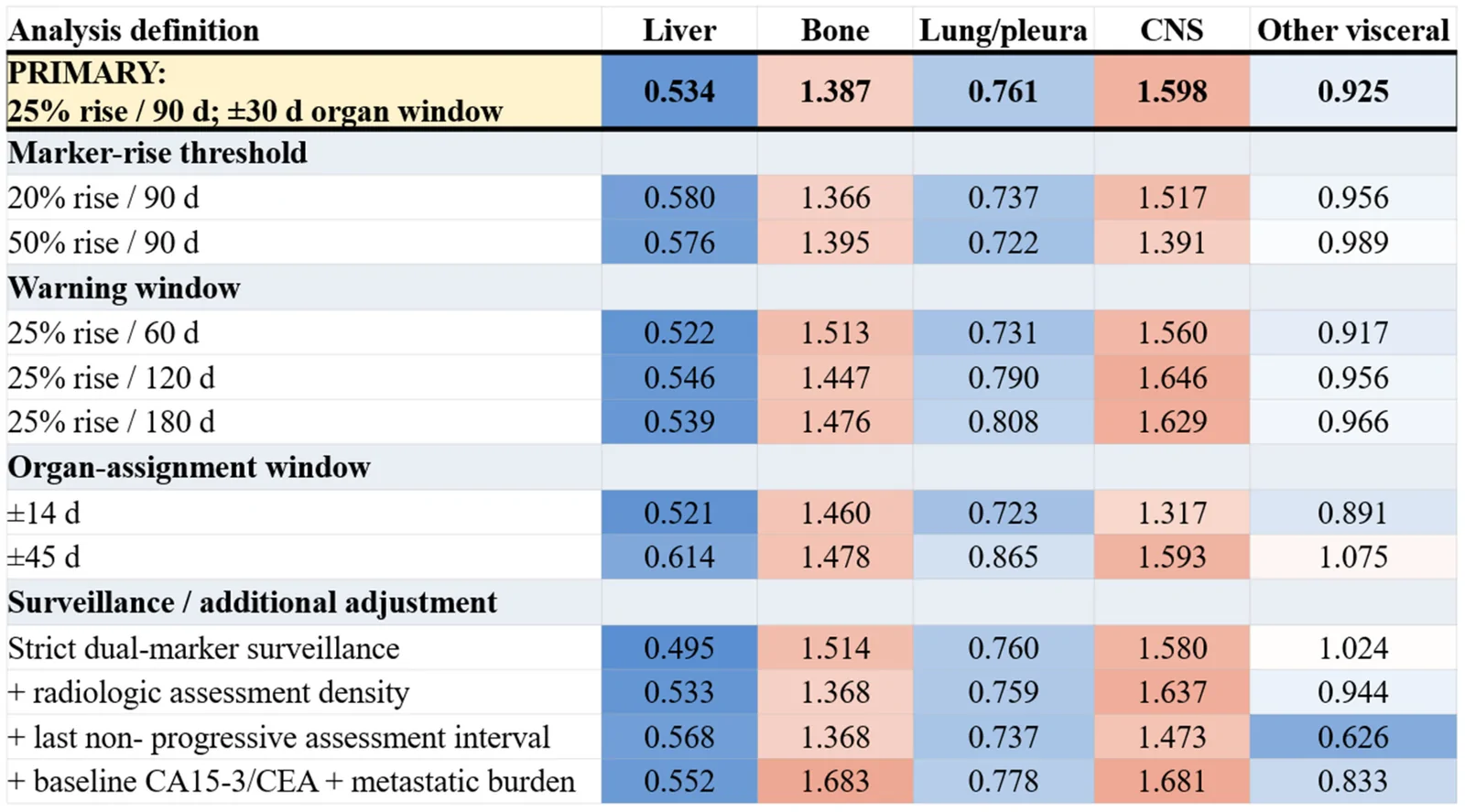
The robustness of the anatomical associations across alternative analysis definitions. A heatmap of the adjusted ORs for progression, without a preceding qualifying CA15-3 or CEA rise across alternative marker-rise thresholds, warning windows, organ-assignment windows, surveillance criteria, and additional adjustment models. The primary analysis (25% rise, 90-day warning window, ±30-day organ-assignment window) is highlighted. Blue indicates OR < 1, white OR = 1, and red OR > 1.

**Figure 5:**
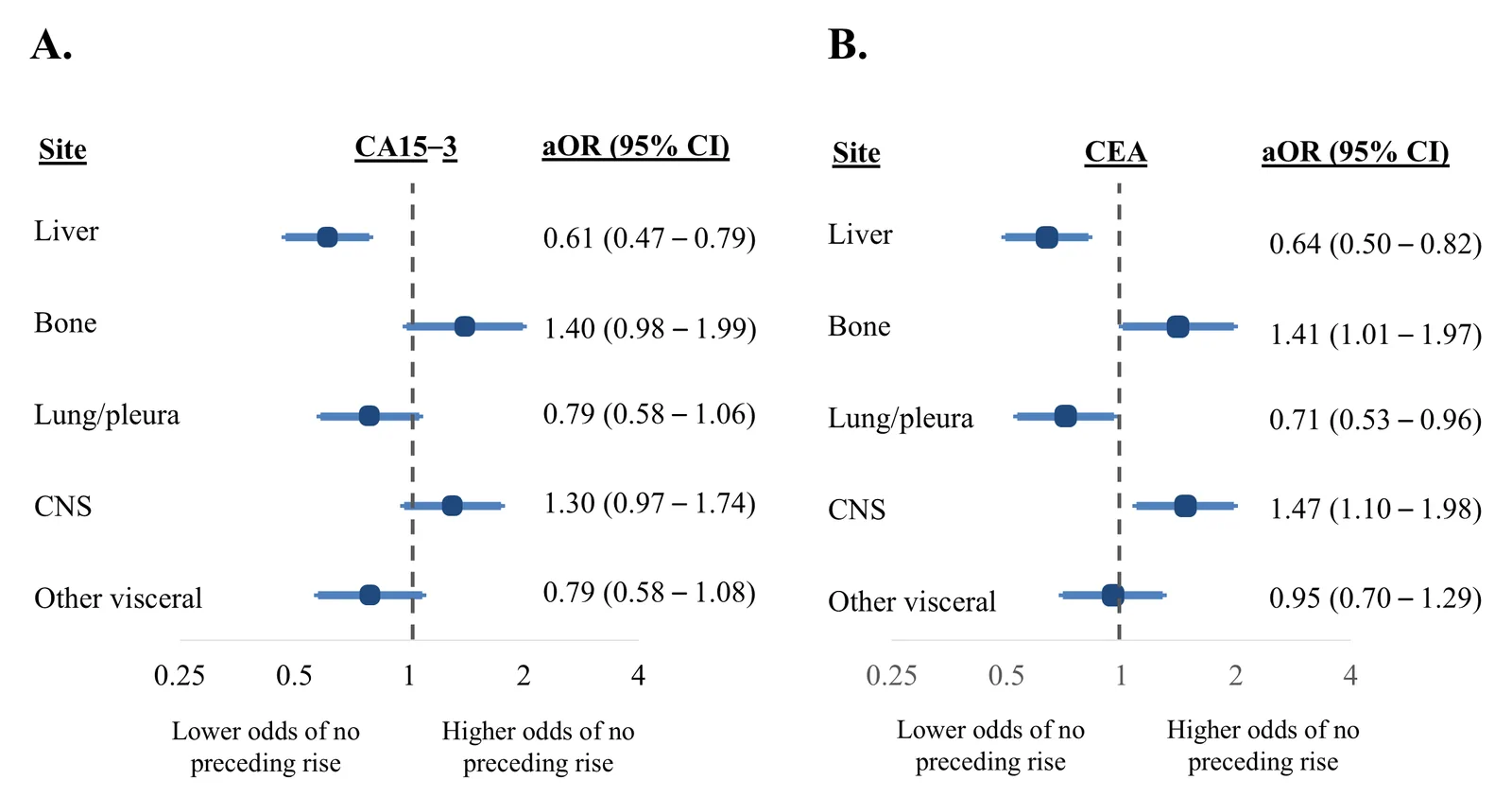
Marker-specific anatomical associations for CA15-3 and CEA. The adjusted odds ratios (aORs) for progression without a qualifying rise in CA15-3 (**A**) or CEA (**B**), estimated using patient-clustered logistic GEE models with the same adjustment framework as the primary analysis. The points indicate aORs and horizontal lines indicate 95% confidence intervals; the dashed line indicates aOR = 1.

**Table 1:** Characteristics of the analysis dataset.

Characteristic	N	Events Without Preceding CA15-3/CEA Rise, n (%)
Primary analytical cohort	4517	1650 (36.5%)
Patients contributing to primary cohort	1592	
Molecular subtype		
HR+/HER2−	3301	1223 (37.0%)
HER2+	753	237 (31.5%)
TNBC	463	190 (41.0%)
Systemic treatment class		
Chemotherapy	2122	842 (39.7%)
Endocrine therapy without CDK4/6	889	281 (31.6%)
ET + CDK4/6 inhibitor	472	152 (32.2%)
HER2-targeted non-ADC	300	102 (34.0%)
ADC	283	98 (34.6%)
Other systemic therapy	451	175 (38.8%)
Analysis/data-availability characteristics		
Both baseline CA15-3 and CEA available	3915	
Last non-progressive imaging interval available	2137	
Strict dual-marker surveillance	3673	
Events with a single newly documented organ site	775	
Events with multiple newly documented organ sites	112	

The data are presented at the progression-event level unless otherwise indicated; individual patients could contribute more than one event. Strict dual-marker surveillance applied the same minimum longitudinal-surveillance criteria as the primary definition, but required at least one measurement of each marker within 30 days before progression. Abbreviations: ADC, antibody–drug conjugate; CA15-3, cancer antigen 15-3; CDK4/6, cyclin-dependent kinase 4/6; CEA, carcinoembryonic antigen; ET, endocrine therapy; HER2, human epidermal growth factor receptor 2; HR, hormone receptor; TNBC, triple-negative breast cancer.

**Table 2:** Qualifying tumor-marker rises and their timing according to newly documented metastatic organ involvement.

Newly Documented Organ Involvement	N Events	Qualifying Rise in Either Marker, n (%)	No Preceding Marker Rise, n (%)	95% CI for Proportion Without a Preceding Rise	Qualifying CA15-3 Rise, n (%)	Qualifying CEA Rise, n (%)	Interval from First Qualifying Marker Rise to Radiographic Progression, Median (IQR), Days
Liver	286	215 (75.2%)	71 (24.8%)	20.2–30.1%	176 (61.5%)	164 (57.3%)	35 (17.0–58.5)
Bone	151	85 (56.3%)	66 (43.7%)	36.1–51.7%	64 (42.4%)	61 (40.4%)	32 (15.0–54.0)
Lung/pleura	194	134 (69.1%)	60 (30.9%)	24.8–37.7%	109 (56.2%)	108 (55.7%)	35 (19.0–57.8)
CNS/brain	206	113 (54.9%)	93 (45.1%)	38.5–52.0%	96 (46.6%)	84 (40.8%)	35 (15.0–56.0)
Other visceral	180	118 (65.6%)	62 (34.4%)	27.9–41.6%	103 (57.2%)	89 (49.4%)	35 (17.3–55.0)

A qualifying marker rise was defined as a ≥25% increase from the preceding running nadir, occurring >30 days after treatment-line initiation and within 90 days before radiographic progression. Organ-site categories were not mutually exclusive. Confidence intervals for the proportions were calculated using the Wilson method; progression intervals were calculated only for events with a qualifying rise. Abbreviations: CA15-3, cancer antigen 15-3; CEA, carcinoembryonic antigen; CI, confidence interval; CNS, central nervous system; IQR, interquartile range.

**Table 3:** The factors associated with progression without a preceding CA15-3/CEA rise in the primary GEE model.

Variable	Adjusted OR (95% CI)	*p* Value	BH-FDR *p*-Value
Anatomical involvement			
New liver involvement	0.53 (0.40–0.71)	<0.001	<0.001
New bone involvement	1.39 (0.98–1.96)	0.063	0.105
New lung/pleural involvement	0.76 (0.55–1.05)	0.094	0.117
New CNS/brain involvement	1.60 (1.19–2.14)	0.002	0.004
New other visceral involvement	0.93 (0.67–1.28)	0.643	0.643
Molecular subtype (reference: HR+/HER2−)			
HER2+	0.73 (0.60–0.89)	0.002	
TNBC	1.11 (0.91–1.37)	0.309	
Treatment class (reference: chemotherapy)			
Endocrine therapy without CDK4/6	0.60 (0.50–0.72)	<0.001	
ET + CDK4/6 inhibitor	0.61 (0.48–0.76)	<0.001	
HER2-targeted non-ADC	0.96 (0.70–1.30)	0.771	
ADC	0.93 (0.71–1.21)	0.570	
Other systemic therapy	0.92 (0.74–1.15)	0.461	
Treatment line (reference: line 1)			
Treatment line 2	1.09 (0.82–1.45)	0.545	
Treatment line 3	0.89 (0.67–1.18)	0.416	
Treatment line 4	1.05 (0.79–1.39)	0.735	
Treatment line 5+	1.03 (0.81–1.30)	0.835	
Observation-process covariates			
The total CA15-3/CEA measurements in the preceding 90 days, per +2 measurements	0.81 (0.75–0.87)	<0.001	
The mean time from the most recent CA15-3/CEA measurements to progression, per +1 week	1.11 (1.07–1.15)	<0.001	

The adjusted odds ratios were estimated using a patient-clustered logistic GEE model, including all five anatomical indicators simultaneously and adjusting for the molecular subtype, treatment class, treatment-line group, marker-measurement frequency, and timing of the most recent marker measurements. BH-FDR correction was applied to the five anatomical coefficients. Abbreviations: ADC, antibody–drug conjugate; BH-FDR, Benjamini–Hochberg false-discovery rate; CA15-3, cancer antigen 15-3; CDK4/6, cyclin-dependent kinase 4/6; CEA, carcinoembryonic antigen; CI, confidence interval; CNS, central nervous system; ET, endocrine therapy; GEE, generalized estimating equations; HER2, human epidermal growth factor receptor 2; HR, hormone receptor; OR, odds ratio; TNBC, triple-negative breast cancer.

## Data Availability

The MSK-CHORD dataset is publicly available through cBioPortal under study identifier msk_chord_2024 (accessed on 1 August 2026) [18].

## Supplementary Materials

The following supporting information can be downloaded at: https://www.mdpi.com/article/10.3390/ijms27198502/s1.

## Abbreviations

ADC, antibody–drug conjugate; aOR, adjusted odds ratio; ASCO, American Society of Clinical Oncology; BH-FDR, Benjamini–Hochberg false-discovery rate; CA15-3, cancer antigen 15-3; CDK4/6, cyclin-dependent kinase 4/6; CEA, carcinoembryonic antigen; CI, confidence interval; CNS, central nervous system; ESMO, European Society for Medical Oncology; ET, endocrine therapy; GEE, generalized estimating equations; HER2, human epidermal growth factor receptor 2; HR, hormone receptor; IQR, interquartile range; LOT, line of therapy; MSK-CHORD, Memorial Sloan Kettering–Clinical History of Real-World Data; OR, odds ratio; TNBC, triple-negative breast cancer; ULN, upper limit of normal.
